# Diabetes in Canadian Women

**DOI:** 10.1186/1472-6874-4-S1-S16

**Published:** 2004-08-25

**Authors:** Catherine Kelly, Gillian L Booth

**Affiliations:** 1 Women's College Hospital, Univeristy of Toronto, 60 Grosvenor Street, Toronto, Canada; 2 St. Michael's Hospital, University of Toronto, 61 Queen Street East, Toronto, Canada

## Abstract

**Health Issue:**

Diabetes mellitus (DM) is a chronic health condition affecting 4.8% of Canadian adults ≥ 20 years of age. The prevalence increases with age. According to the National Diabetes Surveillance System (NDSS) (1998–1999), approximately 12% of Canadians aged 60–74 years are affected. One-third of cases may remain undiagnosed. The projected increase in DM prevalence largely results from rising rates of obesity and inactivity.

**Key Findings:**

DM in Canada appears to be more common among men than women. However, among Aboriginal Canadians, two-thirds of affected individuals are women. Although obesity is more prevalent among men than women (35% vs. 27%), the DM risk associated with obesity is greater for women. Socio-economic status is inversely related to DM prevalence but the income-related disparities are greater among women. Polycystic ovarian syndrome affects 5–7% of reproductive-aged women and doubles their risk for DM. Women with gestational diabetes frequently develop DM over the next 10 years.

**Data Gaps and Recommendations:**

Studies of at risk ethnic/racial groups and women with gestational diabetes are needed. Age and culturally sensitive programs need to be developed and evaluated. Studies of low-income diabetic women are required before determining potential interventions. Lifestyle programs in schools and workplaces are needed to promote well-being and combat obesity/inactivity, together with lobbying of the food industry for needed changes. High depression rates among diabetic women influence self-care ability and health care expenditures. Health professionals need further training in the use of effective counseling skills that will assist people with DM to make and maintain difficult behavioural changes.

## Background

Diabetes is a major cause of heart disease, blindness, kidney failure and limb amputations in Canada and thus contributes a huge economic cost to the Canadian health care system. The National Diabetes Surveillance System (NDSS) estimates that 4.8% of Canadians (1.13 million) aged 20 years and older in the participating provinces had physician-diagnosed diabetes in 1998–1999. [1] It is estimated that as many as one third of cases may remain, as yet, undiagnosed. The number of people with diabetes mellitus (DM) is projected to increase substantially over the next 20 years, largely as a result of increases in rates of obesity and inactivity, as well as the aging of the population.

Native Canadians have a threefold increased risk of impaired glucose tolerance (IGT) and type 2 DM, Aboriginal women being particularly vulnerable. Other ethnic groups that are at increased risk of developing DM include South Asians, Orientals, African Canadians and Hispanics. Although the overall prevalence of DM is slightly higher among men, low-income women are particularly at risk. Further research is needed to determine the cultural groups affected and the other important determinants of health in these women of low socio-economic status.

Determining the true Canadian prevalence rates of DM remains a difficult task. Administrative databases such as the NDSS and Ontario Diabetes Database (ODD) are thought to provide a more comprehensive source from which to assess the population prevalence, as they capture persons who are seen, diagnosed and/or treated with the conditions in the health care system. However, cases of undiagnosed DM and people who do not seek medical attention are not captured in these statistics. In the United States, the data from the third National Health and Nutrition Examination Survey (NHANES) suggest that up to 30% of DM may be undiagnosed. [2] This proportion is felt to be similar in Canada.

Population surveys also attempt to determine the prevalence of diagnosed DM, but self-reporting in population surveys is known to underestimate actual rates of disease in the population. [3-6] Hux found that many people identified as having DM in the ODD, who were known to be taking medication for DM (Ontario drug database), did not indicate that they had diabetes in the Ontario Health Survey (OHS) II. [6] The reasons for this low self-reporting of diabetes are not well understood and are likely complex. The large Canadian surveys have not included certain at-risk people, including people living in chronic care institutions, Native Canadians living on reserves, or people in remote areas of Quebec and Ontario. This omission would also tend to create an underestimate of the true prevalence rates.

## Methods

A review of current literature was undertaken to identify Canadian studies relating to the incidence and prevalence of diabetes and its complications. Studies from countries similar to Canada were reviewed to supplement Canadian data and to provide information where there are gaps in Canadian data. Data based on a variety of sources were used to estimate the prevalence of diabetes, including self-reported survey data and health care utilization administrative databases. Further analyses were conducted using data from the 2000–2001 Canadian Community Health Survey (CCHS). The proportion of respondents with a self-reported diagnosis of diabetes was determined within age, sex, ethnic and socio-economic subgroups. The proportion of women with diabetes first diagnosed during pregnancy was also determined by category of ethnicity. Estimates weighted to the age and sex distribution of the general Canadian population are reported.

## Results

### Prevalence

**Figure 1:**
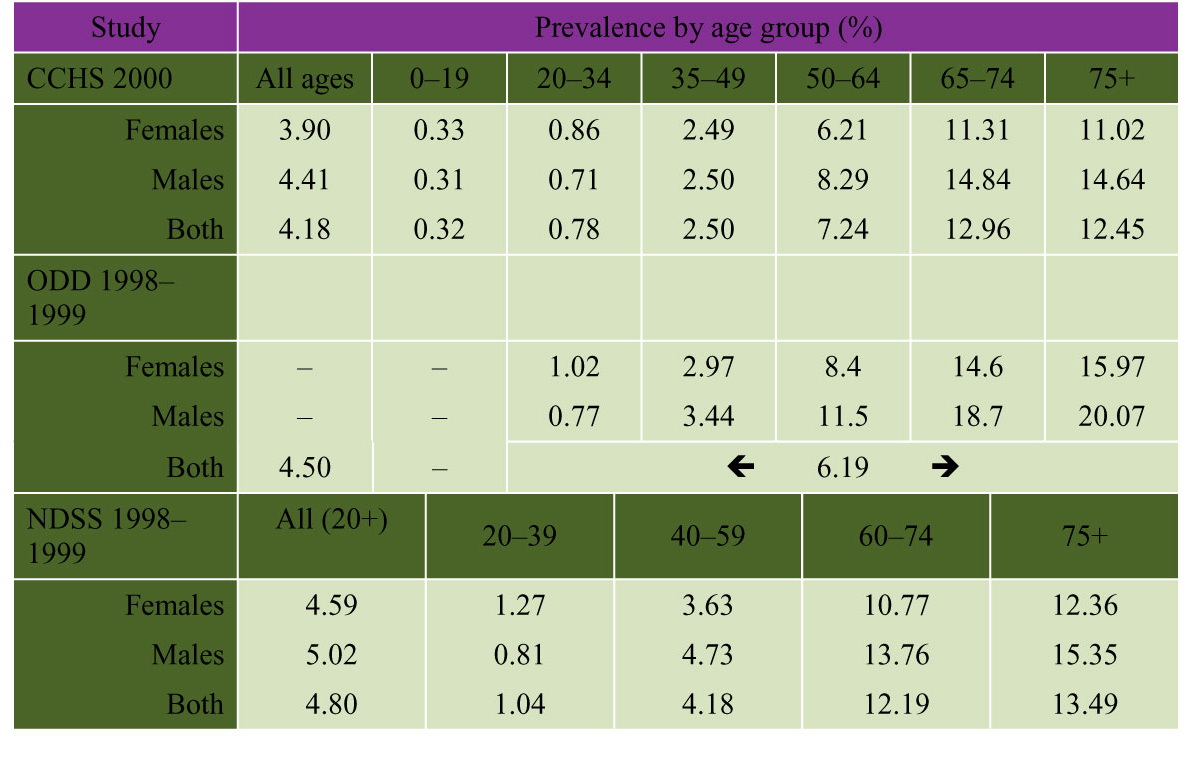
**Prevalence Rates of DM by Age and Sex. **CCHS represents self-reported DM. ODD and NDSS are administrative data, based on diagnosed DM. All prevalence data are age adjusted.

The 1996–1997 National Population Health Survey (NPHS) revealed a prevalence of self-reported, diagnosed DM of 3.2% among Canadians aged 12 and over; the overall prevalence was estimated at 4.9%, including undiagnosed cases of DM. [7,8] The 2000 CCHS found rates of self-reported, diagnosed diabetes that corresponded to an overall prevalence of 4.2% (Figure 1). Rates rose sharply with age in the older age categories, with a prevalence of 13% among those over 65.

From administrative data from the NDSS (1998–1999) [1], the prevalence of diagnosed DM was found to be 4.8% among people over 20 years of age (4.6% of women and 5.0% of men), excluding New Brunswick, Newfoundland and Labrador, Northwest Territories and Nunavut (Figure 1). The NDSS estimates that approximately 1.7 million Canadians had DM (diagnosed and undiagnosed) in 1998–1999. Blanchard developed an administrative database and estimated a 1991 overall prevalence (diagnosed and undiagnosed) of DM of 6.7% among Manitobans aged 25 years and over, representing a 50% rise since 1986. [9] In Ontario, Hux [6] used the ODD and found that the prevalence of diagnosed DM rose from 4.8% in 1994–1995 to 6.2% in 1998–1999 among adults aged 20 and over (Figure 1). Prevalence rates were highest in Toronto and in Ontario counties with large Aboriginal populations, reinforcing the importance of acquiring regional information for planning local health initiatives. [10]

### Impact of Sex on the Prevalence of Diabetes

In most reports, the prevalence of diabetes is marginally higher among males than females, particularly in middle and older age groups (Figure 1). This finding may be due to a greater predisposition towards abdominal obesity and insulin resistance among older men. The only age range in which the prevalence of diabetes is higher among women appears to be the 20 to 34 year category. This may simply represent an increase in case-finding among women of child-bearing age, who have more frequent medical visits. The ODD includes an algorithm to exclude cases of gestational DM (GDM), but the survey data from the CCHS may include some women with GDM, contributing to this increased prevalence. The propensity of women with GDM to develop type 2 DM may also contribute to this finding. There were no sex differences in the prevalence of diabetes among children, who have largely type 1 diabetes.

### Undiagnosed Diabetes

**Figure 2:**
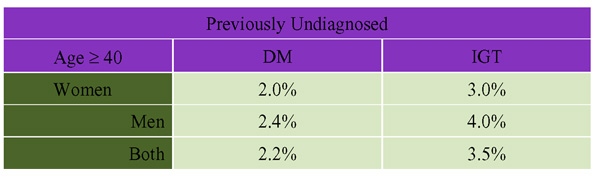
**Prevalence of Previously Undetected Diabetes and Impaired Glucose Tolerance (IGT) Among Primary Care Practices in Canada **(DIASCAN) (Age-Adjusted). Source: Harris et al "The prevalence of NIDDM and associated risk factors in native Canadians" Diabetes Care 1997; 20:185–187. Prevalence of Previously Undetected Diabetes and Impaired Glucose Tolerance (IGT) Among Primary. Care Practices in Canada (DIASCAN) (Age-Adjusted)

The Canadian Diabetes Screening in Canada (DIASCAN) study [11] screened for the presence of undiagnosed diabetes or glucose intolerance among people 40 years of age and older attending family physicians' offices across Canada. Of 9,000 patients who underwent oral glucose tolerance testing, 2.2% had previously undiagnosed DM (Figure 2). The DIASCAN population is not truly representative of the Canadian population over 40 years of age, as the sample includes only those people who are visiting their family physician and are likely to have more illness than other Canadians. This is reflected in the fact that the study population had a diabetes prevalence of 16.4%.

### Incidence

In Manitoba, the 1991 incidence rate of DM among adults 25 years and over was 6.0/1,000 among men and 5.3/1,000 among women. These data, based on an administrative algorithm, estimated the incidence rate to be stable between 1986 and1991. [9] The NPHS 1996–1997 (longitudinal file) gave an incidence rate of 2.6 new cases (95% confidence interval [CI] 2.0, 3.2) per 1,000 people/year aged 12 and over. [12] The U.S. Centers for Disease Control and Prevention reported a similar annual incidence rate in the general population, of approximately 2.9/1,000. [13]

### Risk Factors

#### Ethnicity

##### Aboriginal Canadians

**Figure 3:**
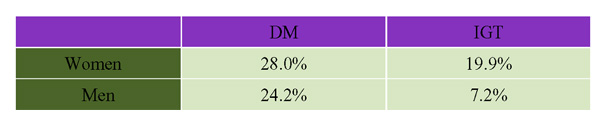
The Prevalence of Diabetes and Impaired Glucose Tolerance (IGT) Among Native Canadians in Sandy Lake, Ontario (Age-Adjusted).

In a 1990 survey, the age-adjusted prevalence rate of self-reported diabetes among First Nations adults on Saskatchewan reserves was 9.7%, as compared with 6.1% among non-First Nations residents of the three Prairie provinces. [7,14] Harris [15] examined the prevalence of type 2 DM and IGT among residents of a remote Native reserve in Sandy Lake, Ontario (Figure 3). Direct testing revealed that females had a higher prevalence of obesity, IGT and type 2 DM, and these conditions tended to occur at younger ages. In a Quebec study, the prevalence of diabetes among female residents of an Algonquin community was noted to be among the highest in the country (49%) and was twice that of male residents (24%). [16] These patterns have been observed throughout Canada, the 1991 Aboriginal Peoples Survey reporting that two thirds of Aboriginal people with diabetes were women. [17]

##### South Asian, Chinese and European Canadians

**Figure 4:**
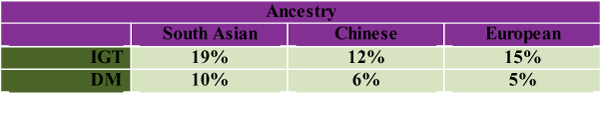
Rates of Diabetes and Impaired Glucose Tolerance (IGT) Among Different Ethnic Groups Participating in the SHARE Study [18] (Age-Adjusted)

The Study of Health Assessment and Risk in Ethnic Groups (SHARE) was a prospective cohort study that examined cardiovascular risk factors and coronary event rates in adults of South Asian, Chinese and European origin living in Hamilton, Toronto and Edmonton. [18] Rates of DM and IGT were highest among South Asians as compared with other ethnic groups (Figure 4). Although sex-specific rates were not reported, some risk factors for DM, such as abdominal obesity, were more common among women of South Asian descent than among women of either Chinese or European ancestry.

**Figure 5:**
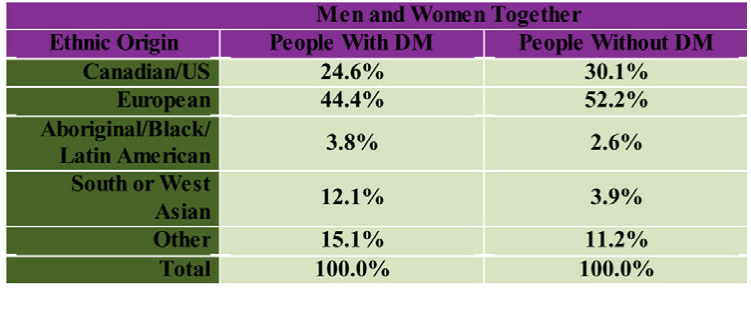
Ethnic Composition of Participants With and Without DM in the Ontario Health Survey [19] (Age-Adjusted)

A higher proportion of participants in the OHS who had DM reported their ethnic origin to be South or West Asian, Aboriginal, Black, Latin American or another non-white category [19] (see Figure 5). In the 2000 CCHS a minority of participants were non-Caucasian (20.7%), and the prevalence of diabetes in ethnic groups considered to be at increased risk (South Asian 4.97%, Aboriginals 4.08%) was much lower than reported in studies where high-risk communities were screened directly (e.g. Sandy Lake data; see Figure 3). This discrepancy may be partly explained by the knowledge that self-report surveys give falsely low estimates of DM prevalence. [4-6]

#### Obesity and Inactivity

Obesity is a major risk factor for the development of DM. Although the prevalence of obesity, defined as a body mass index (BMI) of 30 or greater, is higher among Canadian men than women (35% versus 27%), [20] the risk of DM associated with excess weight is relatively greater for women. [13] An analysis of the OHS II found that obese women were four times as likely to develop DM as lean women, whereas obesity in men was associated with a less than threefold elevation in risk. The risk of diabetes rose with BMI levels above 25 for both sexes. [10]

Sex-related patterns of obesity are different in the Aboriginal population. Several studies have noted a higher rate of obesity among Aboriginal women of all ages. [21,22] Hanley [22] found that the prevalence of overweight among Aboriginal children and adolescents was directly associated with the number of television viewing hours and inversely associated with dietary fibre consumption and fitness. Rates of obesity are substantially higher among Aboriginal than non-Aboriginal children, although rates continue to rise in both groups. Tremblay [23] reported that the prevalence of overweight increased from 15.0% to 29.2% among girls and from 15.0% to 35.4% among boys between 1981 and 1996, and the prevalence of obesity (BMI > 30) tripled among children of both sexes (5% to 14.6% for girls and 5% to 16.6% for boys). Physical inactivity among Canadians of all ages is felt to be the most important factor leading to the dramatic rise in rates of obesity. [24] Excess food and caloric intake also contributes to the rise in obesity. The OHS II found that the risk of DM among women and men was greater for those physically inactive than for those who were active (relative risk [RR] 1.5 and 1.4 respectively)

#### Socio-economic Status

Socio-economic status (SES), measured by either income or educational level, appears to be inversely related to the prevalence of DM. Of women participating in the OHS II, 21.0% of those with DM were in the low-income category in contrast to only 10.4% of women without diabetes. [19] Similarly, the age-adjusted RR of diabetes was 1.61 among women and 1.33 among men who had had less than a high school education, as compared with those who had reached college or university. Data from the 2000 CCHS revealed that people with DM had a substantially lower median household income than did those people without DM. Among women aged 20 to 65, household income under $20,000 was associated with a twofold higher prevalence of DM compared with those with higher levels. Income-related disparities in diabetes prevalence appeared to be greater among women.

Risk factors for developing diabetes, including obesity, sedentary lifestyles and poor nutrition, appear to be more common among lower income groups in both Canada and the United States. [25,26] High rates of immigration of ethnic groups known to have a greater predisposition towards developing diabetes (African, Hispanic or Asian descent) might be another potential explanation, although little research has been done to determine the risk of diabetes among new Canadian immigrants.

### Multiple Risk Factors

The influence of multiple risk factors on the development of diabetes seems to be magnified by whether those at risk are male or female. Data from the OHS 1996–1997 on the prevalence of three risk factors (BMI > 27, physical inactivity, low income) among Ontarians with and without DM illustrate this. The prevalence of all three risk factors among women with DM was 9.5% as compared with 1.9% among women without DM (rate ratio 5.0, p < 0.005); among men with DM, only 2.8% had all three risk factors, as compared with 1.6% among men without DM, a difference that was not statistically significant. The Canadian Heart Health Surveys Research Group [20] found a greater prevalence of physical inactivity among women who had a higher body mass index.

### Gestational Diabetes

Gestational diabetes mellitus (GDM) is defined as glucose intolerance that occurs only during pregnancy. A Toronto-based study found the prevalence of GDM with direct glucose tolerance testing to be 7% among predominantly white women. [27] A retrospective study (1990–1993) of Native Canadians in the Sioux Lookout Zone in northern Ontario reported GDM rates of 8.5%, and rates among Cree women in northern Quebec (1995–1996) are as high as 13%. [28,29] The risk of women with GDM developing type 2 DM within 5 to 16 years after their pregnancy ranges from 17% to 63%. [30-33] The risk is higher among women whose condition is diagnosed before 24 weeks of gestation, among obese women and among those of Hispanic, African and Native ancestry.

Survey data from the 2000 CCHS revealed that a considerably higher proportion (14.1%) of Aboriginal women self-reported a diagnosis of DM during pregnancy than women of other ethnic groups (Caucasians, 5.9%; all other ethnic groups combined, 6.6%).

### Polycystic Ovarian Syndrome

Polycystic Ovarian Syndrome (PCOS) is one of the most common endocrine disorders, affecting 4.7% to 6.8% of women of reproductive age.[29,34,35] It is characterized by hyperandrogenism (acne, seborrhea, hirsutism), chronic anovulation (< 9 menses per year), and an increased propensity to develop DM. The Nurses' Health Study II [36] compared Caucasian women who had normal menstrual cycles with those who had a cycle length of 40 days or more (or cycles too irregular to estimate). Women with long or highly irregular menstrual cycles had a significantly greater risk of developing type 2DM (RR 2.08, 95% CI 1.62, 2.66), and this persisted after adjustment for BMI. Studies reveal that 31% to 35% of women with PCOS have IGT on oral glucose tolerance testing, and an additional 7.5% to 10% have DM. [37-39]

### Impaired Glucose Tolerance

The importance of early identification of impaired glucose tolerance (IGT) is confirmed in the results of two large prospective studies demonstrating that lifestyle changes can delay or reduce the progression to overt diabetes in people with IGT.[40,41] A U.S. study[41] demonstrated that the lifestyle intervention reduced the incidence of DM by 58% over 2.8 years and was similar by sex and ethnic background.

### Impact of Diabetes on Women

#### Depression and Social Context

A 2001 meta-analysis demonstrated that the odds of finding clinically relevant depression among adults with diabetes was twice that among people without diabetes (odds ratio [OR] 2.0, 95% CI 1.8, 2.2). [42] The prevalence of comorbid depression was significantly higher among diabetic women (28%) than diabetic men (18%), just as it is in the non-diabetic population. The OHS 1996–1997 data showed that the prevalence of depression among women with DM was 8.3%, as compared with 5.4% among women without DM; the comparable rates among men were much lower, at 3.6% and 2.5%. [19] Depression in people with diabetes is associated with poor metabolic control and the use of more health care resources, even after differences in age, sex, race/ethnicity, health insurance and comorbidity are adjusted for. [43] There are inadequate data on at-risk ethnic groups. [44] Ethnic and sex differences in knowledge and health beliefs may affect diabetes self-care, as indicated by several small studies. [45,46] Further qualitative research is needed in this area.

#### Long-Term Complications of Diabetes

Diabetes is the most common cause of acquired blindness among adults in North America. [56] Canadian data on visual problems and diabetic retinopathy are sparse. The prevalence of retinopathy at baseline in the U.K. Prospective Diabetes Study [47] was comparable among women (35%) and men(39%). A 2002 study in the United Kingdom estimated the prevalence of blindness and partial sight in the general and diabetic populations (age-standardized), analyzing sex and ethnic differences. There was an almost threefold increase in the prevalence of blindness and partial sight in the diabetic population. [47] Among those with DM, there was a higher proportion of visually impaired females than males and no overall ethnic differences. In the general population, there was a higher than expected proportion of visually impaired Indo-Asian people. The degree to which the visual loss was caused by diabetic retinopathy is not known. In the Ontario OHS 1996–1997, women with and without diabetes had a higher prevalence of visual problems not corrected by lenses than men. [19] The prevalence of visual problems among women with DM (4.4%) was not statistically different from that of women without DM (2.4%).[24] The prevalence of visual problems among men with and without DM was 1.9%and 1.7%.

Retinopathy is a microvascular complication of diabetes, and it is hypothesized that women with myocardial ischemia and normal coronary arteries may have microvascular disease. The U.S. Atherosclerosis Risk in Communities study found that retinal arteriolar narrowing was related to the risk of coronary heart disease (CHD) in women but not in men. [48] Further studies involving women with diabetes are needed for confirmation of this finding.

Cardiovascular disease (CVD) is the leading cause of death among people with diabetes. From the NPHS 1996–1997 data it was estimated that the prevalence of self-reported, diagnosed cardiovascular disease (CHD and stroke) was 21% among people aged over 12 with DM versus 4% among those without DM (age-adjusted)[12]. (These figures have to be interpreted with some caution, since using prevalence data to examine the risk of more fatal conditions such as CVD has some limitations.) Recent analyses based on the ODD showed that DM appears to reduce the difference in heart disease normally observed between men and women. In fact, acute myocardial infarction (MI) rates among women with DM exceeded those among men without DM. [49] In contrast, the effect of DM on the risk of stroke appears to be similar for men and women. [50]

The SHARE cohort study looked at cardiovascular risk and event rates in adults of three ethnic groups (South Asians, Chinese and Europeans) living in Hamilton, Toronto and Edmonton. [51] South Asians had the highest prevalence of cardiovascular disease as compared with Europeans and Chinese (11%, 5%, and 2% respectively, p = 0.004). The rates of death from cerebrovascular disease (CBVD) were relatively low, and there was little ethnic variation among men. Among women, Chinese women had the highest rate of death from CBVD. A five-year chart review of hospital admissions for acute MI of South Asian Canadians at two hospitals within the metropolitan Toronto region revealed that they were more likely to have DM (43%) than patients not of South Asian origin (28%) (p < 0.001). [52] However, South Asian Canadians did not differ from the other groups with respect to blood pressure or lipid status. Paradoxically, fewer South Asians smoked, a finding that would be expected to reduce the prevalence of heart disease. The underlying mechanisms contributing to the increased CHD and diabetes prevalence in the South Asian populations needs further basic research.

Knowledge about sex differences in the development and progression of diabetic nephropathy (kidney disease) in Canadians is limited. One 10-year prospective cohort study of 385 patients with type 2 DM addressed the impact of metabolic control and blood pressure control on the incidence and progression of nephropathy, but found no effect of sex. [53] Two studies have suggested that males were more likely to develop DM nephropathy.[54,55] Further Canadian studies are needed with adequate representation of the ethnic groups at risk for DM, controlling for blood pressure and metabolic control.

### Mortality Rates

In 1996, there were 5,447 deaths (2,701 males; 2,746 females) in which diabetes was stated to be the underlying cause [12] Thus, diabetes ranks as the seventh leading cause of death in Canada. However, many deaths related to the complications of diabetes remain unrecognized, as DM may not be listed on the death certificate. It is estimated that the actual number of deaths attributable to diabetes is five times as great as this figure. The Institute for Clinical Evaluative Sciences, using OHS II 1996–1997 administrative data, estimated that the life expectancy is about 13 years less for both women and men with diabetes than for those without diabetes (women: 70.6 versus 82.9 years; men: 64.7 versus 77.5 years). [19] Overall, mortality rates were twice as high among individuals with diabetes than in the non-diabetic population.

## Discussion

### Data and Knowledge Gaps

The NDSS will address many of the current gaps in the knowledge about disease prevalence and complication rates by establishing standard data collection procedures and obtaining regional data that will assist local areas to target prevention and treatment. However, this methodology relies on the use of administrative data sources and thus cannot replace the need for primary data collection to identify clinical factors that influence diabetes outcomes. There remains a knowledge gap for ethnic and racial groups other than Aboriginal Canadians and Canadians of European ancestry because of insufficient numbers in survey data and inadequate identification of other ethnic groups in administrative data sets. There remains inadequate information about cardiovascular and retinopathy outcomes in women of these other ethnic groups.

Income-related disparities in diabetes prevalence seem to be greater among women; the reason for this remains unclear. Qualitative and quantitative research to study these low-income women with diabetes is needed to determine ethnic and racial differences, social supports, marital status, housing information, family structure and number of dependent children, employment status, lifestyle and activity profiles, dietary habits, health beliefs, and depression. Obtaining more detailed information about these women may help to elucidate factors that could be modifiable and thus potentially improve health outcomes. Depression is almost twice as prevalent among women with diabetes as among those without, and it influences a woman's ability to obtain metabolic control. Research to determine whether more aggressive treatment of depression and/or mood disorders improves diabetes control could have a significant impact on health outcomes.

Further basic research is needed to better understand the pathogenesis of cardiovascular disease in DM, particularly the ethnic and gender differences. Further basic research is also needed to understand the complications of DM and to provide mechanisms for treatment and prevention. Obesity continues to be a major problem requiring both basic science and behavioural research.

### Policy Implications and Recommendations

The rising obesity problem in Canadian children of all ethnic groups is indicative of a huge potential impact on diabetes incidence and prevalence in the future. Policies to promote healthy lifestyle among Canadians of all ages are urgently required. The school systems across the country need to examine the amount of time given to physical education and health promotion, starting in preschool and continuing throughout high school. Many high schools have eliminated compulsory physical education classes (or an alternative activity) early in high school, and this has resulted in less active lifestyles. Government and public pressure to encourage schools and fast food outlets to serve healthier foods in smaller portions should be encouraged. Lifestyle promotion programs for adults, particularly in the workplace, should be studied. Canadians are working longer hours than they did 15 years ago, making it difficult to find adequate time out of the normal work hours for sports or other activities.

It is important to closely study, in adequate numbers, lower-income Canadian women to try to identify and understand the complex factors that may be contributing to the high prevalence of diabetes. These data are required before effective interventions can be determined and studied.

It is also important to study Canadian women of other ethnic groups who have a higher prevalence of diabetes in adequate numbers to determine the course of their diabetes and target culturally sensitive treatment programs. This includes ensuring access to diabetes education programs and other diabetes services for Canadians of all ethnicities and cultures. Aboriginal women are particularly prone to develop obesity and type 2 DM. Culturally sensitive community programs need to be planned, implemented and evaluated.

Women who have gestational diabetes are known to be at significant risk of developing type 2 DM in the future. It seems prudent to target these young women for diabetes prevention programs in their communities and monitor the outcomes of these programs. Large prospective trials have demonstrated that lifestyle interventions are extremely effective in preventing the progression to diabetes.

Canadian health professionals need further training in the use of effective counselling skills that will assist people with diabetes to make and maintain some difficult behavioural changes.

## Note

The views expressed in this report do not necessarily represent the views of the Canadian Population Health Initiative, the Canadian Institute for Health Information or Health Canada.

## References

[B1] Health Canada (2002). Diabetes in Canada Ottawa: Centre for Chronic Disease Prevention and Control, Population and Public Health Branch, Health Canada, Cat H49-121/2002E.

[B2] Harris MI, Eastman RC (2000). Early detection of undiagnosed diabetes mellitus: a US perspective. Diabetes Metab Res Rev.

[B3] Mackenbach JP, Looman CW, van der Meer JB (1996). Differences in the misreporting of chronic conditions, by level of education: the effect on inequalities in prevalence rates. Am J Public Health.

[B4] Kriegsman DM, Penninx BW, van Eijk JT, Boeke AJ, Deeg DJ (1996). Self-reports and general practitioner information on the presence of chronic diseases in community-dwelling elderly. A study on the accuracy of patients' self-reports and on determinants of inaccuracy. J Clin Epidemiol.

[B5] Martin LM, Leff M, Calogne N, Garrett C, Nelson DE (2000). Validation of self-reported chronic conditions and health services in a managed care population. Am J Prev Med.

[B6] Hux JE, Ivis F, Flintoft V, Bica A (2002). Diabetes in Ontario: determination of prevalence and incidence using a validated administrative data algorithm. Diabetes Care.

[B7] Tan H, MacLean DR (1995). Epidemiology of diabetes mellitus in Canada. Clin Invest Med.

[B8] Harris MI, Hadden WC, Knowler WC, Bennett PH (1987). Prevalence of diabetes and impaired glucose tolerance and plasma glucose levels in U.S. population aged 20–74 years. Diabetes.

[B9] Blanchard JF, Ludwig S, Wajda A, Dean H (1996). Incidence and prevalence of diabetes in Manitoba, 1986–1991. Diabetes Care.

[B10] Hux JE, Tang M, Hux JE, Booth G, Laupacis A (2002). Patterns of prevalence and incidence of diabetes. Diabetes in Ontario: an ICES practice atlas.

[B11] Leiter LA, Barr A, Belanger A, Lubin S, Ross SA, Tildesley HD, Fontaine N (2001). Diabetes Screening in Canada (DIASCAN) Study: prevalence of undiagnosed diabetes and glucose intolerance in family physician offices. Diabetes Care.

[B12] (1999). Diabetes in Canada: national statistics and opportunities for improved surveillance, prevention, and control Health Canada, Cat No H49-121/1999.

[B13] Geiss LS, Herman WH, Goldschmid MG (1993). Surveillance for diabetes mellitus – United States, 1980–1989. MMWR.

[B14] Pirro MP, Dyck RF, Gillis DC (1996). Diabetes prevalence rates among First Nations adults on Saskatchewan reserves in 1990: comparison by tribal grouping, geography and with non-First Nations people. Can J Public Health.

[B15] Harris SB, Gittelsohn J, Hanley A, Barnie A, Wolever TM, Gao J, Logan A, Zinman B (1997). The prevalence of NIDDM and associated risk factors in native Canadians. Diabetes Care.

[B16] Delisle HF, Rivard M, Ekoe JM (1995). Prevalence estimates of diabetes and of other cardiovascular risk factors in the two largest Algonquin communities of Quebec. Diabetes Care.

[B17] Health Canada (1997). Medical Services Branch Diabetes among First Nations people: information from the 1991 Aboriginal Peoples Survey carried out by Statistics Canada Ottawa.

[B18] Kohner EM, Aldington SJ, Stratton IM, Manley SE, Holman RR, Matthews DR, Turner RC (1998). UKPDS 30: diabetic retinopathy at diagnosis of NIDDM and associated risk factors. Arch Ophthalmol.

[B19] Manual DG, Schultz SE, Hux JE, Booth G, Laupacis A (2002). Diabetes health status and risk factors. Diabetes in Ontario: an ICES practice atlas.

[B20] Reeder BA, Chan Y, Macdonald SM, Angel A, Sweet L (1997). Regional and rural-urban differences in obesity in Canada. Canadian Heart Health Surveys Research Group. Can Med Assoc J.

[B21] Young TK, Dean HJ, Flett B, Wood-Steiman P (2000). Childhood obesity in a population at high risk for type 2 diabetes. J Pediatr.

[B22] Hanley AJ, Harris SB, Gittelsohn J, Wolever TM, Saksvig B, Zinman B (2000). Overweight among children and adolescents in a Native Canadian community: prevalence and associated factors. Am J Clin Nutr.

[B23] Tremblay MS, Willms DJ (2000). Secular trends in the body mass index of Canadian children. Can Med Assoc J.

[B24] Rabkin SW, Chen Y, Leiter L, Liu L, Reeder BA (1997). Risk factor correlates of body mass index. Can Med Assoc J.

[B25] Choiniere R, Lafontaine P, Edwards AC (2000). Distribution of cardiovascular disease risk factors by socioeconomic status among Canadian adults. Can Med Assoc J.

[B26] Winkleby MA, Kraemer HC, Ahn DK, Varady AN (1998). Ethnic and socioeconomic differences in cardiovascular disease risk factors. Findings for women from the Third National Health and Nutrition Examination Survey, 1988–1994. JAMA.

[B27] Sermer M, Naylor CD, Gare DJ (1995). Impact of increasing carbohydrate intolerance on maternal fetal outcomes in 3637 women: the Toronto Tri-Hospital Gestational Diabetes Project. Am J Obstet Gynecol.

[B28] Harris S, Caulfield L, Sugamori M (1997). The epidemiology of diabetes in pregnant Native Canadians. Diabetes Care.

[B29] Rodrigues S, Robinson E, Gray-Donald K (1999). Prevalence of gestational diabetes mellitus among James Bay Cree women in northern Quebec. Can Med Assoc J.

[B30] Damm P, Kuhl C, Bertelsen A, Molsted-Pedersen L (1992). Predictive factors for the development of diabetes in women with previous gestational diabetes. Am J Obstet Gynecol.

[B31] Metzger BE, Cho NH, Roston SM, Radvany R (1993). Prepregnancy weight and antepartum insulin secretion predict glucose tolerance five years after gestational diabetes mellitus. Diabetes Care.

[B32] Coustan DR, Carpenter MW, O'Sullivan PS, Carr SR (1993). Gestational diabetes: predictors of subsequent disordered glucose metabolism. Am J Obstet Gynecol.

[B33] Kjos SL, Peters RK, Xiang A, Henry OA, Montoro MN, Buchanan TA (1995). Predicting future diabetes in Latino women with gestational diabetes: utility of early postpartum glucose tolerance testing. Diabetes.

[B34] Knochenhauer ES, Key TJ, Kahsar-Miller M (1998). Prevalence of the polycystic ovary syndrome in unselected black and white women of the southeastern United States: a prospective study. J Clin Endocrinol Metab.

[B35] Diamanti-Kandarakis E, Kouli CR, Bergiele AT (1999). A survey of the polycystic ovary syndrome in the Greek island of Lesbos: hormonal and metabolic profile. J Clin Endocrinol Metab.

[B36] Solomon CG, Hu FB, Dunaif A, Rich-Edwards J, Willett WC, Hunter DJ, Colditz GA, Speizer FE, Manson JE (2001). Long or highly irregular menstrual cycles as a marker for risk of type 2 diabetes mellitus. JAMA.

[B37] Legro RS, Kunselman AR, Dodson WC, Dunaif A (1999). Prevalence and predictors of risk for type 2 diabetes mellitus and impaired glucose tolerance in polycystic ovary syndrome: a prospective, controlled study in 254 affected women. J Clin Endocrinol Metab.

[B38] Ehrmann DA, Barnes RB, Rosenfield RL (1999). Prevalence of impaired glucose tolerance and diabetes in women with polycystic ovary syndrome. Diabetes Care.

[B39] Asuncion M, Calvo RM, San Millan JL (2000). A prospective study of the prevalence of the polycystic ovary syndrome in unselected Caucasian women from Spain. J Clin Endocrinol Metab.

[B40] Tuomilehto J, Lindstrom J, Eriksson JG, Valle TT (2001). Prevention of type 2 diabetes mellitus by changes in lifestyle among subjects with impaired glucose tolerance. N Engl J Med.

[B41] (2002). Reduction in the incidence of type 2 diabetes with lifestyle intervention or metformin. Diabetes Prevention Program Research Group. N Engl J Med.

[B42] Anderson RJ, Freedland KE, Clouse RE, Lustman PJ (2001). The prevalence of comorbid depression in adults with diabetes: a meta-analysis. Diabetes Care.

[B43] Egede LE, Zheng D, Simpson K (2002). Comorbid depression is associated with increased health care use and expenditures in individuals with diabetes. Diabetes Care.

[B44] Tellez-Zenteno JF, Cardiel MH (2002). Risk factors associated with depression in patients with type 2 diabetes mellitus. Arch Med Res.

[B45] Brown SA, Harrist RB, Viaagomez ET, Segura M, Barton SA, Hanis CL (2000). Gender and treatment differences in knowledge, health beliefs, and metabolic control in Mexican Americans with type 2 diabetes. Diabetes Educator.

[B46] Sammuel-Hodge CD, Headen SE, Skelly AH, Ingram AF, Keyserling TC, Jackson EJ, Ammerman AS, Elasy TA (2000). Influences on day-to-day self-management of type 2 diabetes among African-American women: spirituality, the multi-caregiver role, and other social context factors. Diabetes Care.

[B47] Hayward LM, Burden ML, Burden AC, Blackledge H, Raymond NT, Botha JL, Karwatowski WS, Duke T, Chang YF (2002). What is the prevalence of visual impairment in the general and diabetic populations: Are there ethnic and gender differences?. Diabet Med.

[B48] Wong TY, Klein R, Sharrett AR, Duncan BB, Couper DJ, Tielsch JM, Klein BEK, Hubbard LD (2002). Retinal arteriolar narrowing and risk of coronary heart disease in men and women: the atherosclerosis risk in communities study. JAMA.

[B49] Booth GL, Rothwell D, Fung K, Tu JV, Hux JE, Booth GL, Slaughter PM, Laupacis A (2002). Diabetes and cardiac disease. Diabetes in Ontario: an ICES practice atlas.

[B50] Kapral MK, Tang M, Rothwell D, Booth GL, Laupacis A, Hux JE, Booth GL, Slaughter PM, Laupacis A (2002). Diabetes and stroke. Diabetes in Ontario: an ICES practice atlas.

[B51] Anand SS, Yusuf S, Vuksan V, Devanesen S, Teo KK, Montague PA, Kelemen L, Yi C, Lonn E, Gerstein H, Hegele RA, McQueen M, for the SHARE Investigators (2000). Differences in risk factors, atherosclerosis, and cardiovascular disease between ethnic groups in Canada: the Study of Health Assessment and Risk in Ethnic groups (SHARE). Lancet.

[B52] Gupta M, Doobay AV, Singh N, Anand SS, Raja F, Mawji F, Kho J, Karavetian A, Yi Q, Yusuf S (2002). Risk factors, hospital management and outcomes after acute myocardial infarction in South Asian Canadians and matched control subjects. Can Med Assoc J.

[B53] Torffvit O, Agardh CD (2001). The impact of metabolic and blood pressure control on incidence and progression of nephropathy: a 10-year study of 385 type 2 diabetic patients. J Diabetes Complications.

[B54] Ravid M, Brosh D, Ravid-Safran D, Levy Z, Rachmani R (1998). Arch Intern Med.

[B55] Gall MA, Hougaard P, Borch-Johnsen K, Parving HH (1997). Risk factors for development of incipient and overt diabetic nephropathy in patients with non-insulin dependent diabetes mellitus: prospective, observational study. BMJ.

[B56] Canadian National Institute of Blindness National Office (2001). CNIB Client Database Toronto.

